# Utilization of *Cladophora glomerata* extract nanoparticles as eco-nematicide and enhancing the defense responses of tomato plants infected by *Meloidogyne javanica*

**DOI:** 10.1038/s41598-020-77005-1

**Published:** 2020-11-17

**Authors:** Rehab Y. Ghareeb, Hanan Alfy, Antwan A. Fahmy, Hayssam M. Ali, Nader R. Abdelsalam

**Affiliations:** 1 Plant Protection and Biomolecular Diagnosis Department, Arid Lands Cultivation Research Institute, The City of Scientific Research and Technological Applications, New Borg El Arab, Alexandria, 21934 Egypt; 2 Plant Protection Research Institute, Field Crop Pests Department, Agricultural Research Center, Giza, 12627 Egypt; 3 Biotechnology Dep, Faculty of Agriculture, Ain Shams University, Ain Shams, 13766 Egypt; 4 Botany and Microbiology Department, College of Science, King Saud University, P.O. Box 2455, Riyadh, 11451 Saudi Arabia; 5 Timber Trees Research Department, Agriculture Research Center, Sabahia Horticulture Research Station, Horticulture Research Institute, Alexandria, 21526 Egypt; 6 Agricultural Botany Department, Faculty of Agriculture (Saba Basha), Alexandria University, Alexandria, 21531 Egypt

**Keywords:** Genetic techniques, Biological techniques, Biotechnology, Genetics, Plant sciences, Environmental sciences

## Abstract

Tomato (*Solanum Lycopersicum* L.) is an important vegetable crop that belongs to the family Solanaceae. Root-knot nematodes reflect the highly critical economically damaging genera of phytoparasitic nematodes on tomato plants. In this study, the eco-nematicide activity of freshwater green macroalga *Cladophora glomerata* aqueous extract and their synthesized silver nanoparticles (Ag-NPs) against root-knot nematodes *Meloidogyne javanica* was investigated on tomato plants. The formation and chemical structure of Ag-NPs was examined. The aqueous extract from *C. glomerata* was applied against the root-knot nematodes besides the biosynthesized green silver nanoparticles with 100, 75, 50, and 25% (S, S/2, S/3, S/4) concentrations. To investigate the plant response toward the Green Synthesized Silver Nanoparticles (GSNPs) treatment, expression profiling of Phenylalanine Ammonia-Lyase (*PAL*), Poly Phenol Oxidase (*PPO*), and Peroxidase (*POX*) in tomato were examined using Quantitative Real-Time PCR (Q-PCR). The results indicated that GSNPs from *C. glomerata* exhibited the highest eco-nematicide activity in the laboratory bioassay on egg hatchability and juveniles (J2S) mortality of *M. javanica* compared with the chemical commercial nematicide Rugby 60%. Also, results showed a significant reduction in galls number, egg masses, females per root system/plant, and mortality of juveniles. The results of *PAL* and *PPO* enzyme expression for the control plants remained relatively stable, while the plant inoculated with nematode *M. javanica* as well as the activity of genes in scope was increased from 14 to 28 Days after Nematode Inoculation (DANI). These activities were improved in inoculated plants and treated with *C. glomerata* extract and their green syntheses of Ag-NPs and the other plants treated with Rugby 60% (4 mL/L). The greatest activities of the three enzymes were evident after 14 days after the nematode inoculation. It can be concluded that the green synthesized nanoparticles using *C. glomerata* could be used as potent nematicides against *M. javanica* which induces the immune system to defend against nematode infection.

## Introduction

Tomato (*Solanum Lycopersicum* L.) is an important vegetable crop grown worldwide in open fields over the year according to the climate conditions. Fungal, bacterial, viral, and nematode diseases cause major production constraints affecting yield and quality^1^. Among various crop-damaging diseases, root-knot nematodes (*Meloidogyne* spp.) represent the greatest vital economically damaging genera of phytoparasitic nematodes on horticultural and field crops by more than 3000 host species^2^. The four common root-knot nematode species, namely *Meloidogyne incognita, M. javanica, M. Arenaria,* and *M. hapla* are the most abundant and damaging nematodes of vegetables^3,4^. The root-knot nematode *M. javanica* causes serious damage in vegetable crops^5^. Plant-Parasitic Nematodes (PPNs) are one of the highly difficult and obstinate pests to manage in crops^6^. Control of plant-parasitic nematodes poses a challenge because of the enormous variety of suitable hosts. Using of chemical toxic nematicides on crops is increasing even though it has been banned by European Union Directives to decrease the contamination of soils and food. Consequently, scientists are studying for alternative low-impact methods of nematode control, for instance, genetic and induced resistance or the usage of biocontrol agents^7–9^.

Biosynthesis of metal and metal oxide nanoparticles exploiting biological agents such as bacteria, fungi, yeast, plant, and algal extracts is becoming more popular in the area of nanotechnology^10–12^. Nowadays, the biological synthesis of metallic nanoparticles is becoming more important as it is reliable and eco-friendly. Metal-synthesized nanoparticles are becoming prominent in recent years because of their remarkable properties and wide range of applications in catalysis^13,14^. The usage of environmentally benign materials like algae for the synthesis of silver nanoparticles presents numerous benefits of eco-friendliness for pesticide purposes due to the absence of toxic chemicals in the synthesis practices. Different techniques are available for the synthesis of silver nanoparticles, for instance, green chemistry approaches^14–16^ and green synthesis of nanoparticles are emerging areas of nanotechnology^17^. Green synthesis proved to be superior to physical and chemical techniques as it is economically feasible, environmentally friendly, and possible to scale up for mass-scale production without any complexity^18^. Green synthesis methods reduce the hazardous, in harmony with global efforts^19,20^. It was revealed that algae-synthesized green nanoparticles were quite stable in solutions, eco-friendly, easily available, and safe to a vast extent because of the wide distribution of the algae. Moreover, the proposed green synthesis method is an advance of bioscience, high yielding, a low-cost technology, and nontoxic to vertebrate animals. Algae are sustainable resources in water ecosystems that are used as food, feed, and medicine^21^. *C. glomerata* is a fresh and marine green alga classified under the Chlorophyceae class, which had been isolated from the AL-Zawraa park in Baghdad -Iraq during October 2018. Methanolic extracts of *C. glomerata* have potential antimicrobial activity^22^.

When pathogens infect plants, the expression of some defense genes is changed to overcome the pathogen attack^23^. Phenylalanine Ammonia-Lyase (*PAL*) is a vital enzyme in the regulation point among primary and secondary metabolism that catalyzes the nonoxidative deamination of phenylalanine to trans-cinnamate^24^. *PAL* gene expression responds to pathogen invasion and numerous abiotic stresses^25^. Also^26^, it was demonstrated that root-knot nematodes generate a series of peroxiredoxins. Peroxiredoxins are located in tissues surrounding the cuticle of J2S and in the hypodermis. Real-time PCR is considered an important technique in molecular plant pathology and has been widely used for quantification of various enzymes’ gene expression activities^27^.

Therefore, the objective of this study is to illustrate the green synthesis of silver nanoparticles using the extract of *C. glomerata*, evaluate the bionematicidal effect of some incorporating botanicals on *M. javanica* infecting tomato plants, and study some of the expressions of defense genes toward treatment with green biosynthesis nematicide.

## Materials and methods

### Chemicals

The commercial pesticide Rugby 60% (Cadusafos 20%) was utilized as a control nematicide, which was purchased from Misr Agriculture Development Company, Egypt, and the silver nitrate (AgNO_3_; 99.99%) was purchased from Votre Partenaire Chimie (SdS), Peypin, France.

### Tomato plants

Susceptible seeds of tomato cultivar “Alisa” were used to determine the nematicidal activity of the green macroalgal extract and their AgNPs (GSNPs) against *M. javanica* under greenhouse condition. The tomato seeds were obtained from the Department of Vegetable Sciences, Faculty of Agriculture, Alexandria University, Alexandria, Egypt.

### Root-knot nematode identification, collection, and preparation

Single-egg masse was used to inoculate tomato plants (*S. Lycopersicum* L.). Seedlings were transplanted in 30 cm diameter plastic pots filled with sterilized clay-sand soil (1:2 v/v) and the pots were maintained at 25 ± 3 °C for 16 h of photoperiod in the experimental greenhouse, the City of Scientific Research and Technological Applications, Alexandria, Egypt. Three months after the infestation of the tomato plant with *Meloidogyne* sp., adult females of root-knot nematode were collected from galled roots for species identification by the characteristics of the perineal pattern technique^28^. Plants uprooted and egg masses were collected from the galls of infected tomato roots with a needle. The roots were cut into small pieces (2–3 cm) and then placed in a flask containing 0.5% NaOCl solution described by^29^. Active juveniles (J2S) of *M. javanica* were obtained by using the Baermann plate technique^30^.

### Collection and preparation of extract by alga material

Samplings were carried out from Mahmoudiyah Canal, El Beheira Governorate, Egypt, at latitude 31.1851°N and longitude 30.5246°E during autumn 2017. Samples of green macroalga were collected manually from the rocks of irrigation drainage that discharges their water directly into the Mahmoudiyah Canal. Samples were collected separately, packed within sealed plastic bags, and transferred directly to the laboratory in the City of Scientific Research and Technological Applications, Alexandria, Egypt. The alga was cleaned carefully with fresh distilled water three times to eliminate any adhering particles such as clay, grass, shells, etc.

The macroalga was dried in the laboratory in a shaded place for 40 days. After completely drying out, the alga materials were ground to a fine powder using an electrical blender. Then, they were weighed and stored in sterilized plastic pages. The preparation of aqueous extracts from algae was done following the method of^31^ with slight modifications. About 10 g of algal fine powder was soaked in 100 mL warm distilled water (Approximately 50 °C) while being shaken at 150 rpm for 72 h. The extract was left to cool off at room temperature and filtered through sterilized cotton gauze. Then filtered through Whatman filter paper no.1 and the filtrate were used as a crude extract.

### Morphological characterization

Morphological variations based on either strain were filamentous; if filamentous strains were found, heterocysts were conducted on them first. The widths and heights of the examined samples were measured by using a light microscope (Olympus BX40 microscope, Japan). Ocular dimensions and images were taken using a Nikon E5000 digital camera in the Biomedical Lab, City of Scientific Research. Taxonomic identifications were performed according to^32,33^.

### Biosynthesis of Ag-NPs using the algal extract

The silver nanoparticles (Ag-NPs) were biosynthesized as described by^34^ with slight modifications. Exactly 10 mL of the previously prepared solution of the aqueous extract of *C. glomerata* extract was slowly dissolved in a 90 mL of AgNO_3_ (1 mM) solution with a magnetic stirrer and heater for the even-coating process of silver. Then, it was exposed to warming at 40 °C for one hour for the reduction of GSNPs, and then flasks were kept in a dark place at 150 rpm for 24 h. The bio-reduction was observed by color converting from pale yellow to dark brown then to dark black. Furthermore, bio-reduction was also measured via UV–Vis spectra at a wavelength of 480 nm on UV–Vis spectroscopy (model T60-PG instruments). The dark black solid product was collected through centrifugation at 10,000×*g* for 10 mins and meticulously washed with fresh distilled water two times with ethanol absolute. The final product was left in an oven at 35 °C overnight. The dried sample was ground into powder and stored on drops of ethanol absolute for further analysis.

### Analysis of Ag-NPs

Ag-NPs synthesized by *C. glomerata* (GSNPs) were examined using an X-ray diffractometer “model (XRD-7000, Shimadzu, Japan” in the City of Scientific Research and Technological Applications lab, Alexandria, Egypt). The Cu-Kα X-rays of wavelength 1.54060 Å was assessed and data were carried for 2θ at a range of 5° to 80° with a step of 0.026°. The chemical structures of silver nanoparticles were examined using FT-IR Spectrometer (FTIR-8400S, Shimadzu, Japan). The silver nanoparticles were recorded in a range from 400 to 4000 cm^–1^ using KBr powder for obtaining the composition of these silver nanoparticles. A Scanning Electron Microscopy (SEM) test was used to illustrate the changes in color in the bionanoparticles and their morphology, using 8000 xg centrifugation (15 min at 4 °C), as described by^35^. The average particle size of the bionano silver ranged from 25 to 55 nm. Assessment of the size, shape, and state of GSNPs were done according to^34^.

### Egg hatchability and juvenile mortality bioassay of *M. javanica *in vitro

An in vitro experiment was conducted to study and determine the efficacy of freshwater macroalgal (*C. glomerata*) aqueous extract and their Green Synthesized Silver Nanoparticles (GSNPs) 10 mg /100 mL on the hatchability of eggs and J2S mortality percentage in the root-knot nematode (*M. javanica*). Egg masses were taken with a needle then transferred to distilled water for three days to hatch, and juveniles were collected. For the egg hatchability bioassay, ten well microtiter tissue culture plates of nematode eggs suspension containing about 50 eggs/200 µL and approximately ten freshly hatched J2S/200 µL per well were analyzed for the mortality bioassay experiment. All treatments of *Cladophora* aqueous extracts and GSNPs were inoculated with nematode eggs for eggs hatchability or freshly hatched J2S for the mortality bioassay. For each experiment, one treatment was left untreated to serve as a check (control) treatment (6 mL d.H_2_O + 200 µL nematode suspension). Also, another treatment was only treated with the nematicide Rugby 60% (6 mL d.H2O + 200 µL nematode suspension + 12 µL Rugby 60%). The other treatments (5) were divided into the following: *Cladophora* aqueous extract 6 mL + 200 µL nematode suspension, eggs for egg hatchability and J2S for mortality bioassay, and (S, S/2, S/4, and S/10) of GSNPs where.

- S = 6 mL GSNPs + 200 µL nematode suspension,
- S/2 = 3 mL GSNPs + 3 mL d.H2O + 200 µL nematode suspension (eggs for eggs hatchability and freshly hatched J2S for mortality bioassay),
- S/4 = 1.5 mL GSNPs + 4.5 mL d.H2O + 200 µL nematode suspension (eggs for eggs hatchability and freshly hatched J2S for mortality bioassay),
- S/10 = 0.6 mL GSNPs + 5.4 mL d.H2O + 200 µL nematode suspension (eggs for eggs hatchability and freshly hatched J2S for mortality bioassay.

Each treatment was repeated five times while the experiment was repeated twice. The numbers of a live J2S were counted using a light microscope after 12, 24, and 48 h intervals from exposure to room temperature. After treatment, juveniles were moved to plain water. Nematodes were considered alive if they moved or maintained a winding shape^36^, while they were considered dead if they did not regain movement after being moved to tap water and probed with a fine needle^37^. The reduction percentage in nematode parameters was calculated according to the following equation:

$$\left( {Reduction} \right) \, \% \, = \frac{Total \;number \;of\; J2S\; in\; control - No. \;of \;alive \;J2S \;in\; treatment}{{Total\; number \;of \;J2S \;in\; control }}*100$$

### Greenhouse experiment

Surface-sterilized seeds of tomato (*Solanum Lycopersicum L.* cv. Alisa) were developed in seedling trays filled with sterilized soil. They were watered daily and fertilized weekly (N/P/K) via a soil-drench solution. Healthy and uniform seedlings were transplanted on plastic pots of 20 cm diameter filled with 2.5 kg/cm^2^ mixture of sterilized sand and soil (1:1). Four thousand freshly hatched *M. javanica* J2S in 2 mL water was introduced into the mixture via four 5-cm-deep holes and were incubated for 45 day at 27 ± 5 °C in the greenhouse. Ten pots were inoculated with nematode and untreated with *C. glomerata* extract or GSNPs to serve as a control. Another ten were inoculated with nematode and treated with Rugby 60% at 1 mL/pot to serve as the second control. The other 20 pots were divided into two groups of ten pots each^38^, with slightly modifications. The first group was inoculated with nematodes (4000 J2S) and treated with *C. glomerata* aqueous extract 50 mL/pot (5 mg/50 mL). The second group was inoculated with nematode and treated with GSNPs at S (100%) concentration. All treatments were applied while the soil was being drenched in 100 mL water/pot immediately with inoculation. The tomato plants were maintained in the greenhouse in the same conditions described previously in a randomized complete block design with 10 cm between each pot for 60 days. The temperature in the greenhouse during the experiment was 25 ± 5 °C, with ten replicates (pots) where the experiment was conducted twice. Plants were harvested 60 days after inoculation. The roots were completely washed with running tap water from surrounding soil and the following parameters were taken: the fresh and dry weights of roots shoots of each plant were measured. *M. javanica*-inoculated roots were soaked in exactly a 0.015% phloxine B solution for 15 min to stain the egg masses^28^ before counting them. Afterward, the *M*. *javanica* eggs were extracted from the tomato roots as in the method provided by^29^ and counted via stereoscopy.

The egg masses number was considered as the infective ability of the nematode because it shows the number of J2S that were able to penetrate, infect the root tissue, and develop into egg-laying females. The number of galls and egg masses per root was calculated as well as the female per root. The number of J2S in 250 g soil/pot was extracted and recorded^39^.

Tomato plants inoculated with *M. javanica* and treated with *C. glomerata* extract or GSNPs were compared with the two control groups and were evaluated by determining the three antioxidant enzymes via qualitative and quantitative approaches. Phenylalanine Ammonia-Lyase (*PAL*), Poly Phenol Oxidase (*PPO*), and Peroxidase (*POX*) were assessed in tomato roots after 0, 3, 7, 14, 28, and 56 Days Post Inoculation (dpi). Five plants per treatment were randomly collected at each specific sampling time, rinsed with demineralized water, and stored in mineralized water after 0, 3, 7, and 14 days. Afterward, they were used for the quantification of gene expression at the same time. One gram of root was homogenized in a 5 ml extraction buffer (50 mM phosphate buffer, pH 7.0, 1 mM EDTA, and 2% Polyvinylpyrrolidone, PVPP) for *POX* and *PPO* extracted and (25 mM Tris-HCl buffer) (PH 8.8) for *PAL* extracted in an ice-cold pastel mortar. The homogenate was centrifuged at 12,000×*g* for 300 min at 4 °C (Ultracentrifuge CFC Free, Gallenkamp, Germany) and the supernatant was directly used for enzyme activity assays^38^. *PAL*, *POX,* and *PPO* activity assays were performed as described by Hussey et al.^29^. For peroxidase activity assay, 0.1 mL of enzyme extract was incubated with 1.5 mL of 0.05 pyrogallol and 0.5 mL of 1% H2O2. The change in the absorbance was recorded at 20 s interval for three minutes at 420 nm (Beckman, Germany). Enzyme activity was expressed as the increase in the absorbance (Δ OD420) min^−1^ g^−1^ of Fresh Weight (FW)^38^. Phenylalanine ammonia-lyase activity was performed as described by Lisker et al. (1983) with a slightly modified procedure. *PAL* activity was determined spectrophotometrically by the production of *trans*-cinnamic acid from L-phenylalanine where the reaction mixture contained 1 mL of enzyme extract, 0.5 mL substrate 50 m M l-phenylalanine, and 0.4 mL 25 mM Tris- HCl buffer. After incubation for two hours at 40 °C. The activity was stopped by the addition of 0.06 ml 5 N HCl, and the absorbance was read at 290 nm against the same volume of reaction mixture without l-phenylalanine, which served as a blank. The enzyme activity was expressed as µmol of trans-cinnamic acid(t-CA)/mg protein/h. Polyphenol oxidase enzyme activity was determined by adding 200 μL of enzyme extract to 1.5 mL of 0.1 M phosphate buffer (pH 6.5) and 200 μL of 10 mM catechol. The changes in absorbance were recorded for one minute at 495 nm. Enzyme activity was presented as ΔOD495 min^−1^ g^−1^ FW^38^. Each experiment had five replicates and was repeated twice. Besides, the root tissue RNA by Quantitative Poly Chain Reaction (Q-PCR) was quantified at the ending of the experiment. The roots were washed trice with fresh sterilized distilled water for ten seconds each and then they were dried onto sterile tissue paper. Total RNA was extracted from tomato roots samples (infected with *M. javanica* and treated with *C. glomerata* extract and their Green Silver Nanoparticles (GSNPs) with 100% (S) concentration at 0, 3, 7, 14, 28, and 56 DANI (Days After Nematode Inoculation) using TRIzol reagent (Invitrogen, USB Cooperation, Cleveland, Ohio, USA), according to the manufacturer’s protocol.

Root tissues were ground well to a fine powder in liquid nitrogen with autoclaved, sterilized, and a pre-cooled porcelain mortar and pestle. About 100 mg of root tissue from each treatment was exposed to RNA extraction, and the resultant RNA was dissolved in 30 µL RNAase free water. RNA integrity was checked on via ethidium bromide stained with 1.5% w/v agarose gel, and the purified total RNA concentration was determined by measuring absorbance at 260 and 280 nm using Spctrostar Nano (BMG LABTECH GmbH/Germany).

For cDNA synthesized from total RNA with oligo (dT) primer, dNTPS, and Moloney-Murine Leukemia Virus Reverse Transcriptase enzyme (M-MLV, RT, Fermentas, USA), according to the standard protocol and buffer (5X), 3 µL of RNA was added to (10 µL (5 ×) RT—buffer), 2.5 µL (25 mM) dNTPs, 5 μL from oligo (dT) primer (20 pmL/μL), 0.25 µL (20 u/µL) RT enzyme, and 4.3 µL H_2_O. RT-PCR amplification was performed in a thermal cycler (Eppendorf, Germany). The mixture was incubated at 40 °C for an hour. Then, it was incubated at 70 °C for ten minutes, followed by storage at − 20 °C until used. Q-PCR was carried out on the resulting cDNA using a Light Cycler Rotor-Gene 6000 system instrument (Qiagen, United States) with a set of gene-specific primers. Phenylalanine Ammonia-Lyase (*PAL*), Polyphenol Oxidase (*PPO*), and Peroxidase (*POX*) and the tomato actin gene were used as internal controls as listed in Table 1.

**Table 1:** The sequence of gene-specific primers, Phenylalanine Ammonia-Lyase (*PAL*), Polyphenol Oxidase (*PPO*), and Peroxidase (*POX*) used in the real-time PCR, and the tomato actin gene which was used as an internal control in the current study.

Primer name	Forward primer (5′–3′)	Reverse primer (5′–3′)	Primer reference
Polyphenol oxidase (*PPO*)	CATGCTCTTATGAGGCGTA	CCATCTATGGAACGGGAAGA	^40^
Peroxidase (*POX*)	GCTTTGTCAGGGGTTGTGAT	TGCATCTCTAGCAACCAACG	^41^
Phenylalanine Ammonia lyase (PAL)	GAGGTGGACAAGGTGTTCGT	TTGCCACAACCCACAACTAA	^42^
Actin (*ACT*)	GAAGGAATAACCACGCTCAG	ACACAGTTCCCATCTACGAG	^43^

The final volume of each reaction 25 μL consisted of 12.5 μL of 2 × Quantitech SYBRGreen RT Mix, forward and reverse primers at 1.5 μL of 10 pmol/μL, 2 μL of template cDNA (50 ng), and 7.5 μL of RNase free water. Reactions were performed in pairs using the following thermal cycling conditions: initial denaturation step at 95 °C for ten minutes. The real-time PCR program included pre-denaturation at 95 °C for 20 min before applying 45 cycles of 95 °C for 15 s, 60 °C for 30 s, and then final extension at 72 °C for 30 s. Relative gene expression at 0, 3, 7, 14, 28, and 56 Days After Nematode Inoculation (DANI), relative to control condition (0 days) were calculated based on the following equations: ^Δ^C q = C q – reference gene, ^ΔΔ^C q = C q – control, and ^ΔΔ^C q expression = 2 (^−ΔΔ^Cq)^44^. Three biological replicates were analyzed.

### Statistical analysis

Data collected in vitro and pot experiments were analyzed by the two-way analysis of variance (treatments and times) using SAS version 9.4 (SAS Institute Inc., Cary, NC, USA). Treatment means were separated by Duncan’s multiple range test at 5% probability.

Significant differences among the means of gene expression and enzyme activity were determined by using Duncan’s multiple range tests (P ≤ 0.05) and were carried out using with SPSS software.

## Results

### Morphological observations

**Figure 1:**
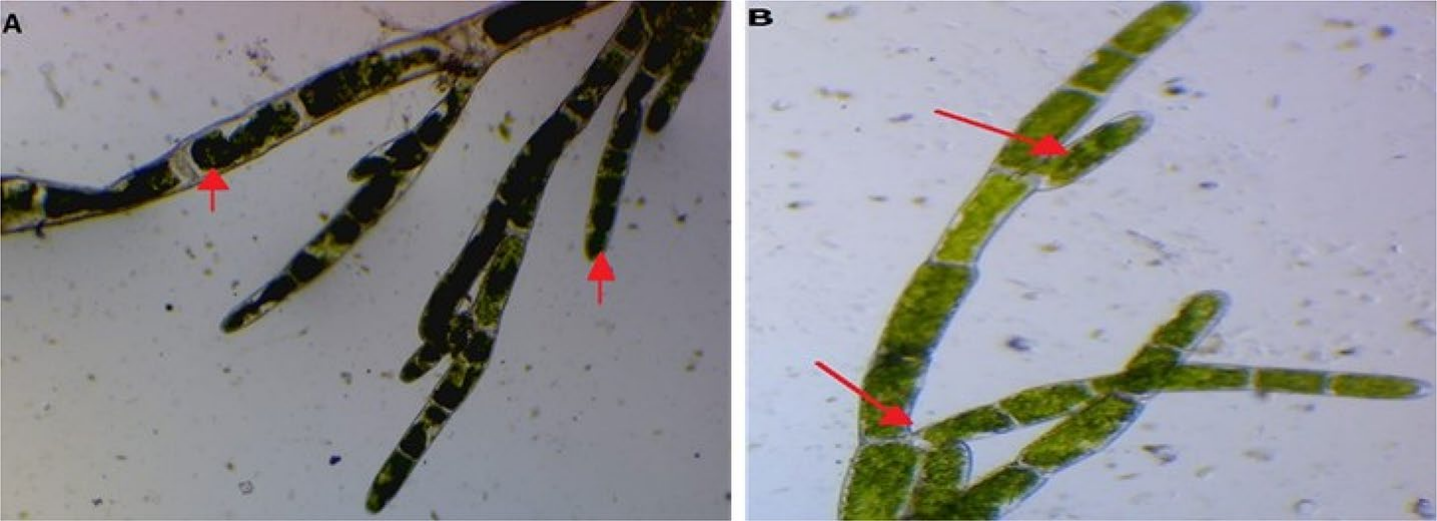
Morphological characteristics of *C. glomerata* (**A**, **B**).

The preliminary identification according to morphological characters observed under light microscopy following green alga strain was purified and their identifications were made. *C. glomerata* is green or dark green, filamentous in form, and attached on rock or cobblestones in the bottom of shallow Channels and watersheds. Thalli are composed of joined cylindrical cells with lengths of 6–20 μm and widths of 4–10 μm and with dichotomously branching filaments. Branches are tufted, arising singly, and arbuscular. Cell walls are thick and usually lamellate. The chloroplast is in a parietal network with numerous pyrenoids. Usually, it tends to maintain a singular spot, which makes it easy to remove (Fig. 1A,B). Morphological characteristics of *C. glomerata* clarified that algal filament was branched chlorophyte with large cylindrical cells forming long, regularly branched structures.

### UV–Vis spectral analysis (*Cladophora glomerata*)

**Figure 2:**
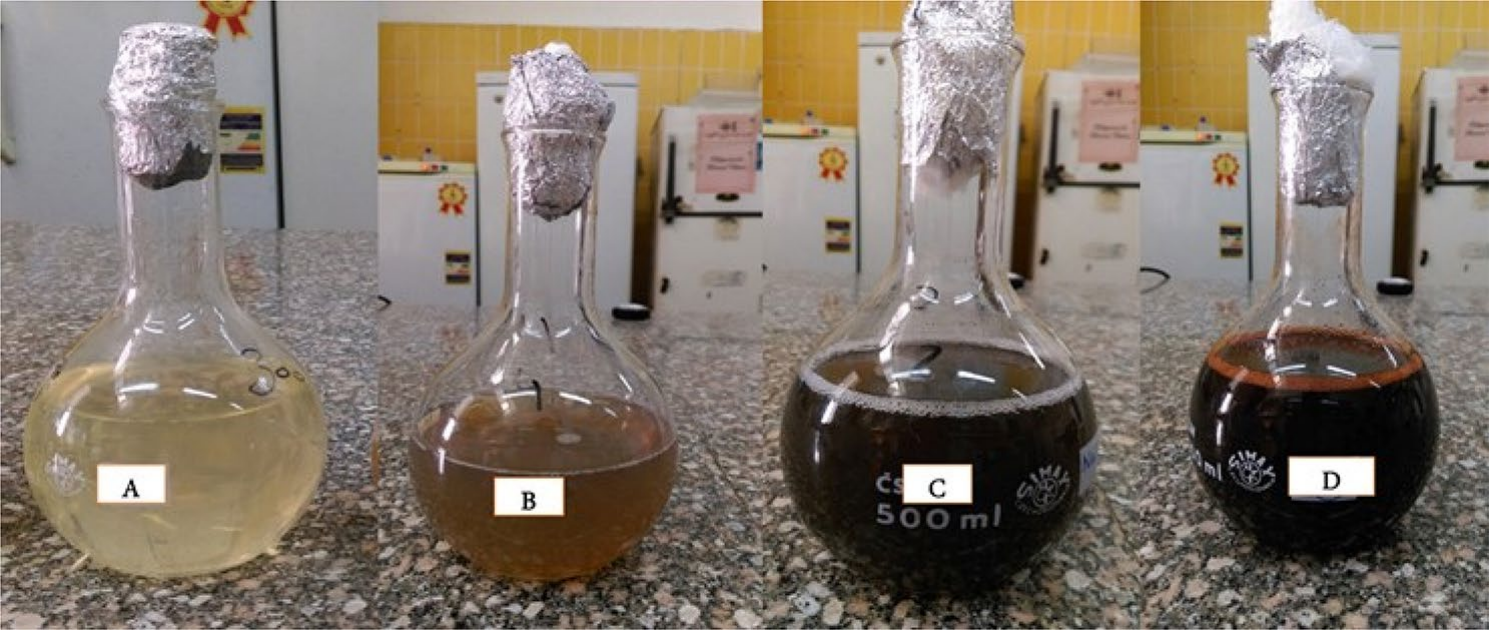
Color change during the bioreduction of AgNO_3_ into Ag-NPs using *C. glomerata* extract: (**A**) before synthesis, (**B**) AgNO_3_ solution after adding algal extract, (**C**) synthesized silver nanoparticles in dark brown color solutions after one hour, and (**D**) synthesized silver nanoparticles in dark black color solutions after three hours.

It is well-established that silver nanoparticles manifest in brown color. In our present study, the silver nanoparticles were illustrated by exposure of *C. glomerata* extract to the AgNO_3_ solution. In aqueous solutions, silver nanoparticles showed clear yellowish, brown, dark brown, and black dark colors by stimulation of surface plasmon vibrations within the particles. The entire reduction of Ag ions was palpable at 1–2 h incubation. After two hours, the color variation was visually observable in the *C. glomerata* extract while incubated with AgNO3 solution in a covered flask (Fig. 2).

### Characterization of silver nanoparticles

**Figure 3:**
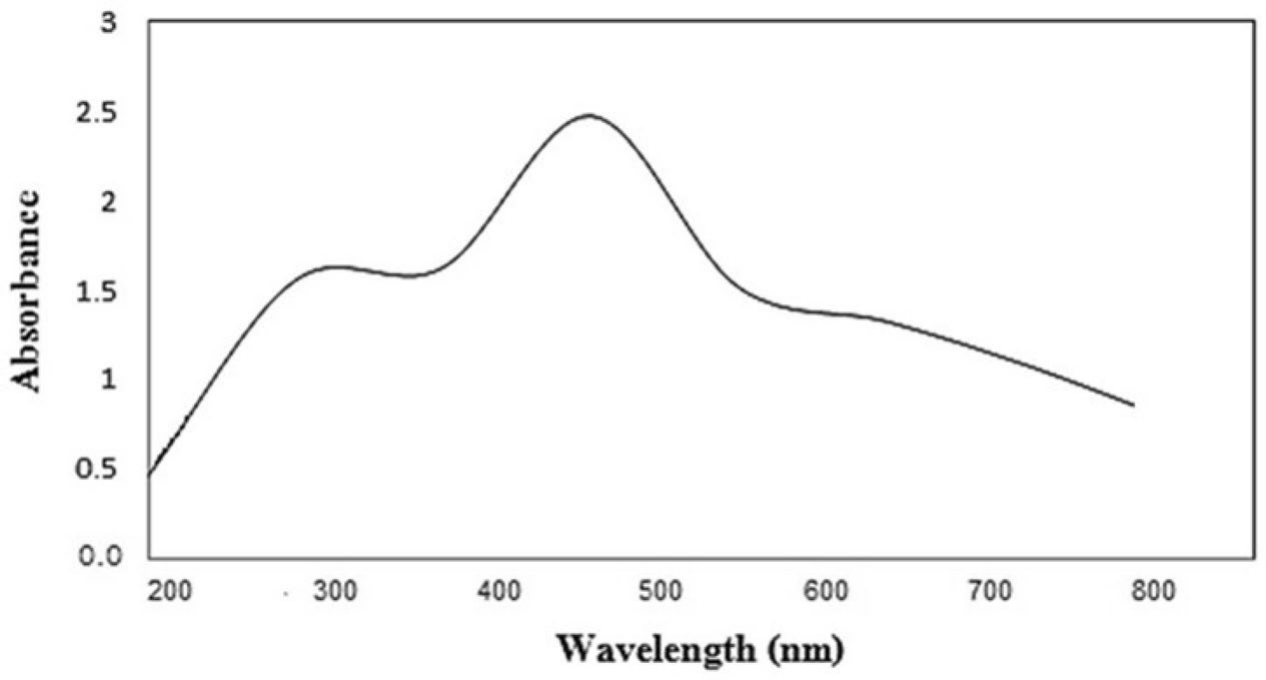
UV–Vis spectra of AgNPs biosynthesized by *C. glomerata* extract at 480 nm.

The formation of silver nanoparticles was confirmed by visual assessment. The UV–vis spectra of silver nanoparticles biosynthesized by *C. glomerata* extract was clarified in Fig. 3. A distinct peak was observed at 480 nm in the UV–vis absorption spectrum (Fig. 3). This absorbance peak indicated a Surface Plasmon Resonance (SPR).

### Characterization of SNPs by XRD

**Figure 4:**
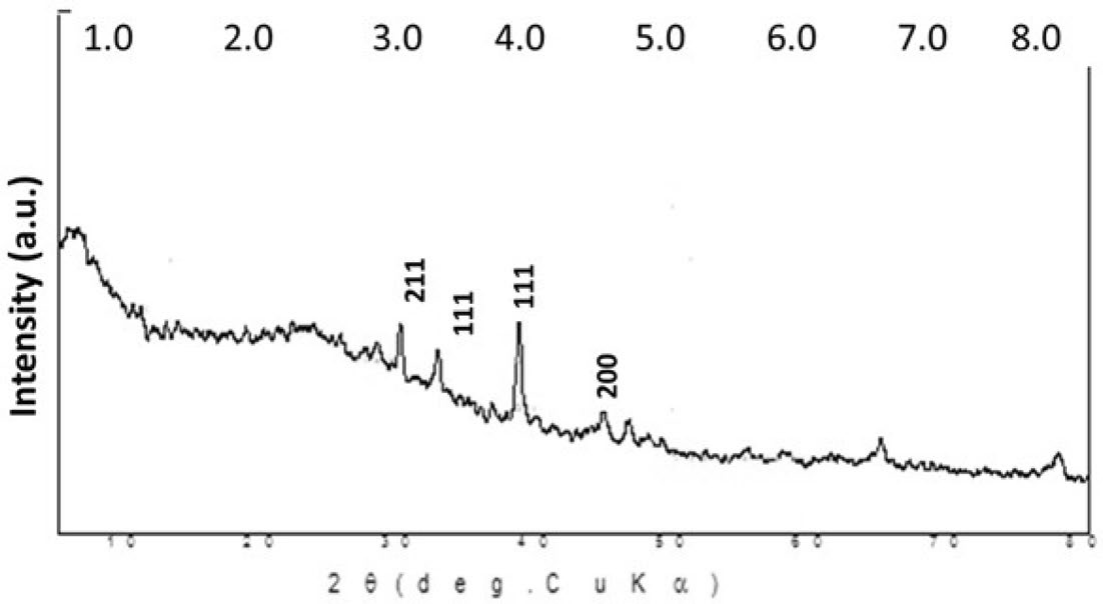
X-ray diffraction pattern of biosynthesized Ag-NPs by *Cladophora glomerata* extract.

X-ray diffraction analysis of dry powders is used to determine the phase distribution, crystallinity nature, and purity of the green synthesized nanoparticles (SNPs) (Fig. 4). Four distinct and vital characteristic peaks were observed at two hours of 29.81, 32.56, 38.47, and 44.65 that correspond to 211, 111, 111, and 200 planes, respectively. Peak 29.81 was indexed to the orthorhombic AgNO_3_ phase and Peak 32.56 was indexed to the cubic Ag_2_O phase. Finally, Peaks 38.4766 and 44.655 were indexed to the cubic structure of the Ag phase with space group which is in good agreement with those of powder silver obtained from the International Center of Diffraction Data’s card (JCPDS, File No. 4-0783). The average crystal sizes of the silver crystallites were calculated from the full width at half-maximum of the diffraction peak using Debye–Scherrer’s equation. The size of the crystallite in different planes of silver was determined with the mean value of all peaks at 13.2 nm.

### FT-IR spectra

**Figure 5:**
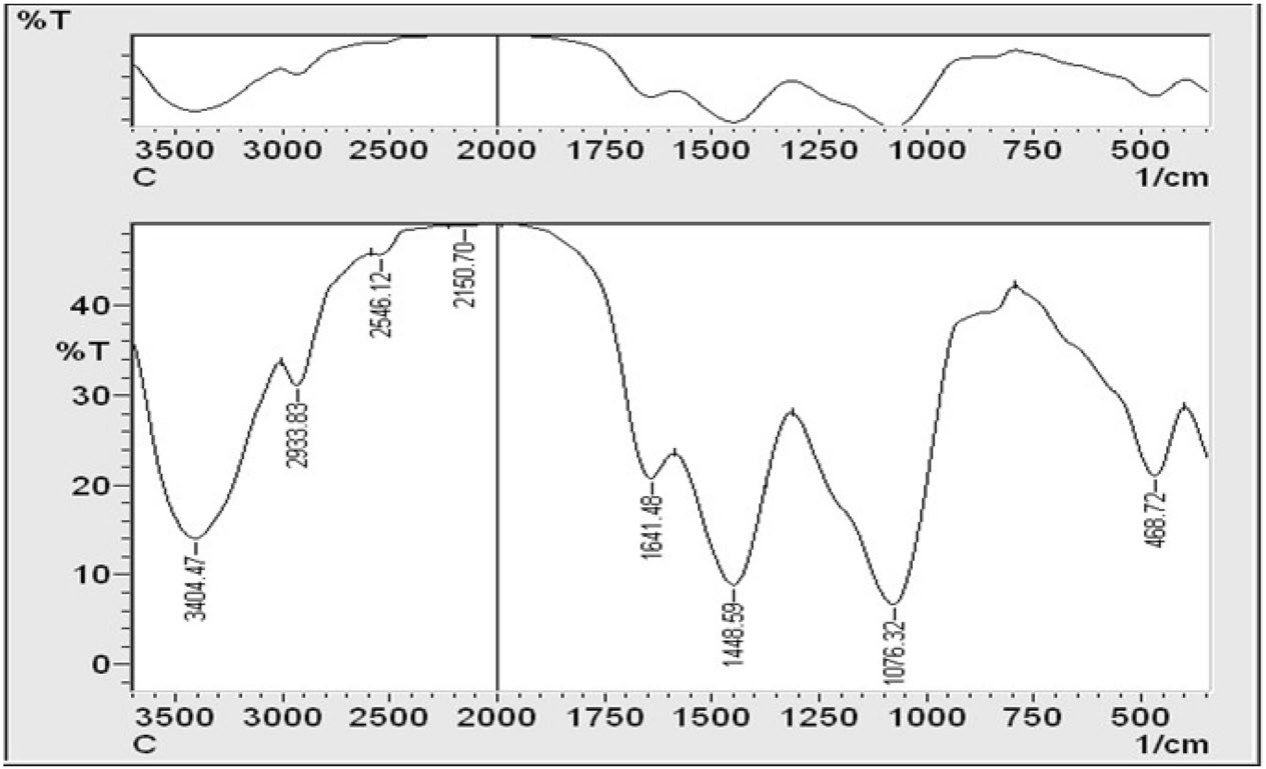
FT-IR spectral pattern of biosynthesized Ag-NPs using *Cladophora glomerata* extract ranging within *3500–500 cm*^*-1*^.*T%* Transmittance%.

The FT-IR spectrum was used to characterize the possible biomolecules responsible for the reduction of the silver ions of the *C. glomerata* biosynthesized nanoparticles Ag-NPs (GSNPs). Figure 5 shows the presence of prominent and distinct peaks: 3404, 2933, 2546, 2150, 1641, 1448, 1076, and 468. The signal at 3404 cm^−1^ displays a strong board O–H stretching vibration carboxylic bands, indicating the presence of H–bonded alcohols and phenols. These absorption frequencies are assigned to the OH group of the algal polysaccharides.

**Figure 6:**
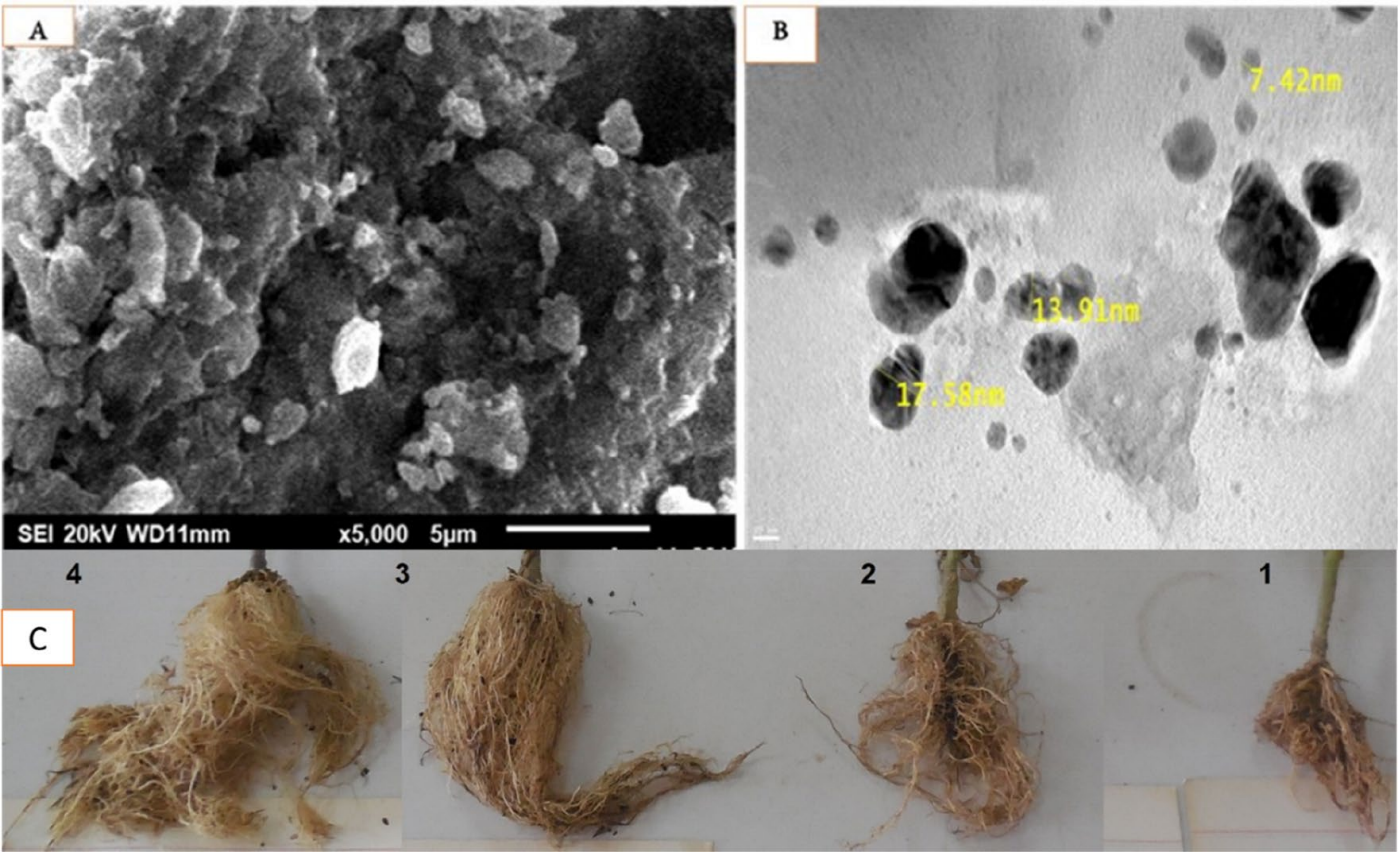
(**A**) Representative high magnification Scanning Electron Microscopy (SEM) micrograph of synthesized cubic and spherical-shaped Ag-NPs and (**B**) Representative high magnification Transmission Electron Microscopy (TEM) micrograph of green synthesized Ag-NPs from *C. glomerata extract* and (**C**) showing the effect of the treatments on the root galls (1) control inoculated with *M. javanica*, (2) control inoculated with *M. javanica* and treatment with Rugby (3) inoculated with *M. javanica* and treated with green synthesized Ag-NPs from *C. glomerata* extract (4) Healthy plant (control without any treatment).

The peak at 2933 cm^−1^ reveals the presence of C–H stretching vibration, indicating the presence of alkanes or assigned to the secondary amine. Another peak at 2546 indicates the SH group, and the peak at 2150 cm^−1^ indicates the presence of –C≡C– group derived from alkynes that are present in the *C. glomerata* biosynthesized nanoparticles Ag-NPs. The peak at 1641 cm^−1^ can refer to the amide groups of protein or the carbonyl stretching groups of the algal polysaccharides. The bands appearing at 1448 cm^−1^ reveal the presence of a C–H bend, indicating the presence of alkanes. 1076 cm^−1^ corresponds to the S–O stretch of the sulfated polysaccharides or reveals the presence of C–N stretching of an aromatic amine group, and, finally, the peak at 468 cm^−1^ is due to Si–O–Si and Al–O–Si bending vibrations. Consequently, the FT-IR results imply that the Ag-NPs were successfully synthesized and capped with biocompounds present in the *C. glomerata* extract by using a green method. Hence, the spectrum of *C. glomerata* aqueous extract was consistent with a sulfated polysaccharide, which is related to its antioxidant activity. To study the morphologies of biosynthesized SNPs, the SEM analysis of colloidal was spherical and hexagonal form Ag-NPs were observed, and it was found to be within 15–50 nm range in sizes and some of them were in the form of agglomerates. GSNPs were observed as deposition on *C. glomerata* extract (Fig. 6). The morphology and size details of the formed biosynthesized nanoparticles from *Cladophora glomerata* extracts were characterized and represented by the Transmission Electron Microscope (TEM) micrograph of silver nanoparticles (Fig. 6). It is evident from the micrograph that individual silver nanoparticles, as well as a number of aggregates, are present and they are spherical and cubical with the maximum diameter sizes of 7.42, 13.9, and 17.5 nm for those prepared from the extracts of *C. glomerata.*

### Nematicidal activity of *C. glomerata* extract and their GSNPs on *M. javanica* under in vitro conditions

In vivo study of the nematicidal activity of the *C. glomerata* aqueous extract and their syntheses of bionanoparticles (GSNPs) were tested against the J2S of the root-knot nematode. *M. Javanica* with different concentration (S = 100%, S/2 = 50%, S/3 = 75%, and S/4 = 25%) were compared with commercial nematicide (Rugby 60%). The data in Table 2 clarified that all treatments decreased the hatchability of *M. javanica* eggs by 89.5–100% compared with the negative control (*M. javanica* + dH_2_O alone) after 24 h. The maximum reduction in hatchability was recorded by the treatment GSNPs with 100; of which 75% was the same. Hence, there were 86.4 and 100 concentrations after 12 and 48 h, respectively. The other treatments were ordered in a descending pattern as follows; Rugby 60% SC, GSNPs with 50%, GSNPs with 25%, and *C. glomerata* aqueous extract only. However, a 100% reduction was recorded after 48 h of treatment.

**Table 2:** The effects of *C. glomerata* extract and their GSNPs on hatchability (H) of *M. javanica* eggs (Mj) after 12, 24, and 48 h of exposure.

Treatment	Exposure time						
	Con. %	12 h		24 h		48 h	
		H	Reduction (%)	H	Reduction (%)	H	Reduction (%)
*M. javanica* + dH_2_O	–	4.4^a^ ± 0.26	–	9.5^a^ ± 0.23	–	24.7^a^ ± 0.25	–
*M. javanica* + Rugby 60	–	0.7^b^ ± 0.025	84.1	0.5^b^ ± 0.025	95	0.0^b^	100
*M. javanica* + *C. glomerata* Extract	100	1.0^b^ ± 0.058	77.3	1.0^b^ ± 0.058	89.5	0.0^b^	100
*M. javanica* + GSNPs	100	0.6^b^ ± 0.018	86.4	0.0^b^	100	0.0^b^	100
	75	0.6^b^ ± 0.018	86.4	0.0^b^	100	0.0^b^	100
	50	0.9^b^ ± 0.032	79.5	0.6^b^ ± 0.009	94	0.0^b^	100
	25	0.99^b^ ± 0.009	77.5	1.0^b^ ± 0.012	89.5	0.0^b^	100

Data are means of five replicates; means are stated with the same letter(s) in each column and are not significantly different at (P ≤ 0.05). (R) Reduction (%) = [Total number of incubated eggs – total number of hatched eggs in treatment]/total number of incubated eggs] × 100.

The effects of *C. glomerata* extract and their silver nanoparticles (GSNPs) on mortality of J2S% (M) of *M. javanica* (Mj) after 24 h, 48 h, and a week were clarified in Table 3. Among all the treatments, the concentrations 100% and 75% of green nanoparticles which proved to induce mortality to juveniles followed by commercial nematicide (Rugby 60%), *C. glomerata* extract, and green nanoparticles 50% and 25%, respectively. The percentages of mortality of J2S of *M. Javanica* were 90.4, 89.5, 87.3, 85.9, 85.7, and 80.9% in 100 and 75% GSNPs, Rugby 60%, *C. glomerata* extract, and 50% and 25% of green nanoparticles, respectively, as compared with control after 12 h. In terms of mobilization of juveniles, out of 100 J2S used in each experiment, 9.6, 10.5, 12.7, 14, 14.3, and 19% larvae remained active in 100 and 75% green nanoparticles, Rugby 60%, *C. glomerata* extract, and 50% and 25% of green nanoparticles, respectively. Similar results remained after 24 h of exposure among all the treatments. The concentrations 100 and 75% of green nanoparticles induced 100% mortality followed by Rugby with 96% mortality, and 93 and 90.3% mortality by concentrations 50 and 25% of GSNPs. Finally, *C. glomerata* extracts induced 89% as compared with control after 24 h of exposure. Likewise, the percentage mortality of J2S was 100% in all treatments and 91 and 94% with the treatment of *C. glomerata* and GSNPs 25%, respectively, as compared with control after 1 week of exposure. In general, percentage mortality was proportionally correlated with the concentrations and exposure periods of extracts. The highest mortality of 100% was recorded in 100 and 75% concentrations of GSNPs at 48 h of exposure time.

**Table 3:** The effects of *C. glomerata* extract and their silver nanoparticles (GSNPs) on mortality (M) of *M. javanica* eggs (Mj) after 12, 24, and 48 h of exposure.

Treatments	Con. %	Exposure time					
		12 h		24 h		48 h	
		Live	Mortality *%*	Live	Mortality *%*	Live	Mortality *%*
*M. javanica* + dH_2_O	–	6.3^a^ ± 0.58	–	7.2^a^ ± 0.85	–	14.2^a^ ± 0.72	–
*M. javanica* + Rugby 60	–	0.8^b^ ± 0.06	87.3	0.3b^c^ ± 0.06	96	0.0^b^	100
*M. javanica* + *C. glomerata* Extract	S	0.9^b^ ± 0.06	85.9	0.8^b^ ± 0.06	89	1.3^b^ ± 0.64	91
*M. javanica* + GSNPs	S	0.6^b^ ± 0.06	90.4	0.0^c^	100	0.0^b^	100
	S/2	0.66^b^ ± 0.06	89.5	0.0^c^	100	0.0^b^	100
	S/4	0.9^b^ ± 0.06	85.7	0.5b^c^ ± 0.06	93	0.0^b^	100
	S/10	1.2^b^ ± 0.06	80.9	0.7^c^ ± 0.06	90.3	0.9^b^ ± 0.20	94

Data are means of five replicates; means with the same letter(s) in each column are not significantly different at P ≤ 0.05. (M) Mortality % = [(Total number of J2S in control − No. of alive J2S in treatment)/No. of Total J2S in control] × 100.

### The efficacy of *C. glomerata* extract and their GSNPs against *M. javanica* (pot experiment)

The data of Table 4 showed the effect of *C. glomerata* extract and GSNPs compared with Rugby 60% on the numbers of nematode galls (G), Egg Masses (EM)/ tomato plant infected with *M. javanica* (Mj), the numbers of nematode females, and the number of juveniles (J2S)/200 g soil after 60 days. Results indicated that all treatments (Mj + *C. glomerata* extract, Mj + GSNPs with concentration 75%, and the nematicide) reduced the number of galls and EM of *M. javanica* over control by 83–93.4% and 93.5–96% compared with the negative control, respectively. Whereas the number of females was reduced with the S/2 (75%) of GSNPs and nematicide treatment by 89, 80, and 87% reductions, respectively. However, data indicated that the numbers of J2S/ 250 g soil were reduced with all treatments by a 93.7–97.3% reduction. The effects of *C. glomerata* extracts, GSNPs, and Rugby 60% SC on growth parameters of tomato plants infected with *M. javanica* (Mj) after 60 days of nematode inoculation were recorded in Table 5. Treatment with *C. glomerata* extracts, GSNPs (S/2), and Rugby 60% caused a positive effect on plant growth by enhancing the plant length (shoot and root) by the centimeter and fresh shoot and root weight by the gram. The maximum plant length was achieved where GSNPs was used with 82.5 cm/shoot and 35.9 cm/root in the same treatment compared with the positive control. GSNPs treatment also produced the highest weight of tomato plants with 24.4 g/shoot and 10.27 g/root. The data compared with the positive control was illustrated in Table 5 and Fig. 6C.

**Table 4:** The effect of *C. glomerata* extract and their silver nanoparticles on the numbers of nematode galls, egg masses, females/root system, and the number of second-stage juveniles (J2S)/250g soil tomato plant infected with *M. javanica* (Mj) after 60 days.

Treatment	Con	Nematode parameters and the decrease in galls over control (D%)							
		Galls	D %	Egg Masses	D %	Females/Root	D %	J_2_S/250 gm Soil	D %
*M. javanica* Alone	–	178^a^ ± 5.8	–	154.2^a^ ± 2.4	–	165.5^a^ ± 5.2		185^a^ ± 6.4	–
*M. javanica* + Rugby 60%	–	11.8^c^ ± 0.6	93.4	8.1^c^ ± 0.6	95	21.24^c^ ± 07	87	10.0^b^ ± 0.6	94.6
*M. javanica* + *C. glomerata* Extract	–	29.4^b^ ± 0.7	83.0	10.2^b^ ± 0.7	93.5	33^b^ ± 0.6	80	13.0^c^ ± 0.6	93.7
*M. javanica* + GSNPs	S	12.2^c^ ± 0.6	93.1	6.2^d^ ± 0.6	96	18.3^d^ ± 0.7	89	5.0^d^ ± 0.5	97.3

Data are means of ten replicates; means with the same letter(s) in each column are not significantly different at (P ≤ 0.05).

**Table 5:** The effect of *C. glomerata* extract, Rugby 60%, and GSNPs on some growth parameters of tomato plants infected with *M. javanica* (Mj) after 60 days.

Treatment	Con	Growth parameters			
		Fresh shoot		Fresh root	
		Length (cm)	Weight (g)	Length (cm)	Weight (g)
Untreated Inoculated Control	–	62.5^c^ ± 0.58	20.0^c^ ± 0.64	23.9^bc^ ± 0.55	8.78^d^ ± 0.12
*M. javanica* + Rugpy 60%	–	58.7^d^ ± 0.34	19.2^c^ ± 0.13	22.7^c^ ± 0.67	8.17^c^ ± 0.74
*M. javanica* + *C. glomerata* Extract	S	71.3^b^ ± 0.52	22.4^b^ ± 0.45	25.0^b^ ± 0.88	9.80^b^ ± 0.45
*M. javanica* + GSNPs	S	82.5^a^ ± 0.19	24.4^a^ ± 0.53	35.9^a^ ± 0.18	10.27^fa^ ± 0.41

Data are means of ten replicates; means with the same letter(s) in each column are not significantly different at (P ≤ 0.05).

### Enzymes activities

**Figures 7:**
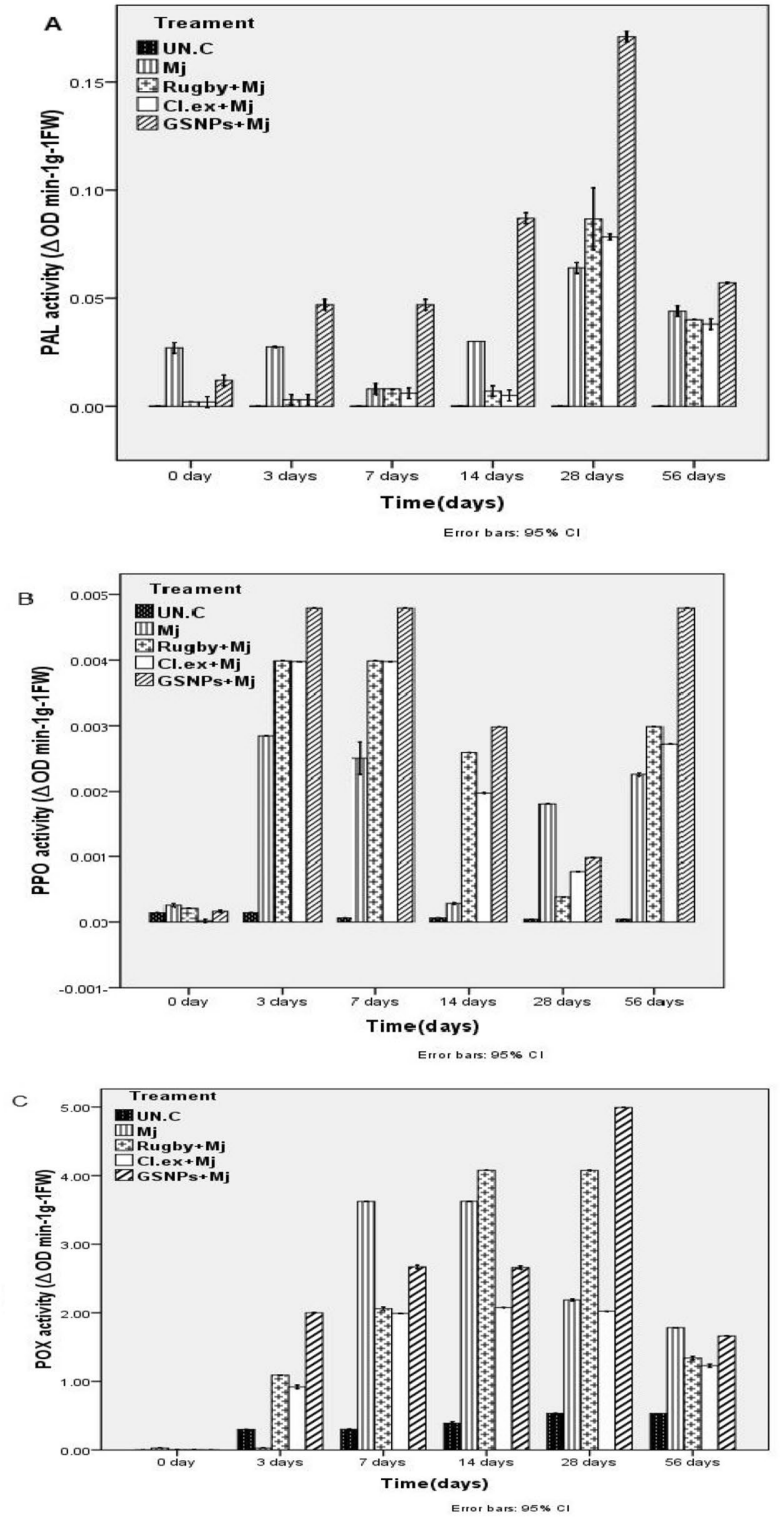
Activities of (**A**) Phenylalanine Ammonia-Lyase (*PAL*), (**B**) Poly Phenol Oxidase (*PPO*), and (**C**) Peroxidase (*POX*) enzymes in the roots of tomato plants.

The results illustrated in Fig. 7 shows that Peroxidase (*POX*), Polyphenol oxidase *(PAL)*, and (*PPO*) enzyme activities of the control plants remained relatively stable, while the inoculation with a nematode (*M. javanica*) significantly increased the activities during 28 to 56 DANI. These activities were uncommonly increased in inoculated plants treated with GSNPs, Rugby nematicide, and extract of *C. glomerata,* respectively. *POX* and *PAL* efficacy for plants treated with GSNPs started to increase significantly and gradually at 3 DANI, where it reached the highest values at 28 DANI. Then, it began to reduce at 56 DANI but still maintained a relatively higher level than that of 0 and 3 DANI (Fig. 7A,C). While the *PPO* activity for plants treated with GSNPs began to increase significantly at 3 and 7 DANI. Then, it began to reduce gradually at 14 and 28 DANI, and then it rises again, reaching its highest value at 56 DANI (Fig. 7B).

### Real-time PCR (QPCR)

**Figure 8:**
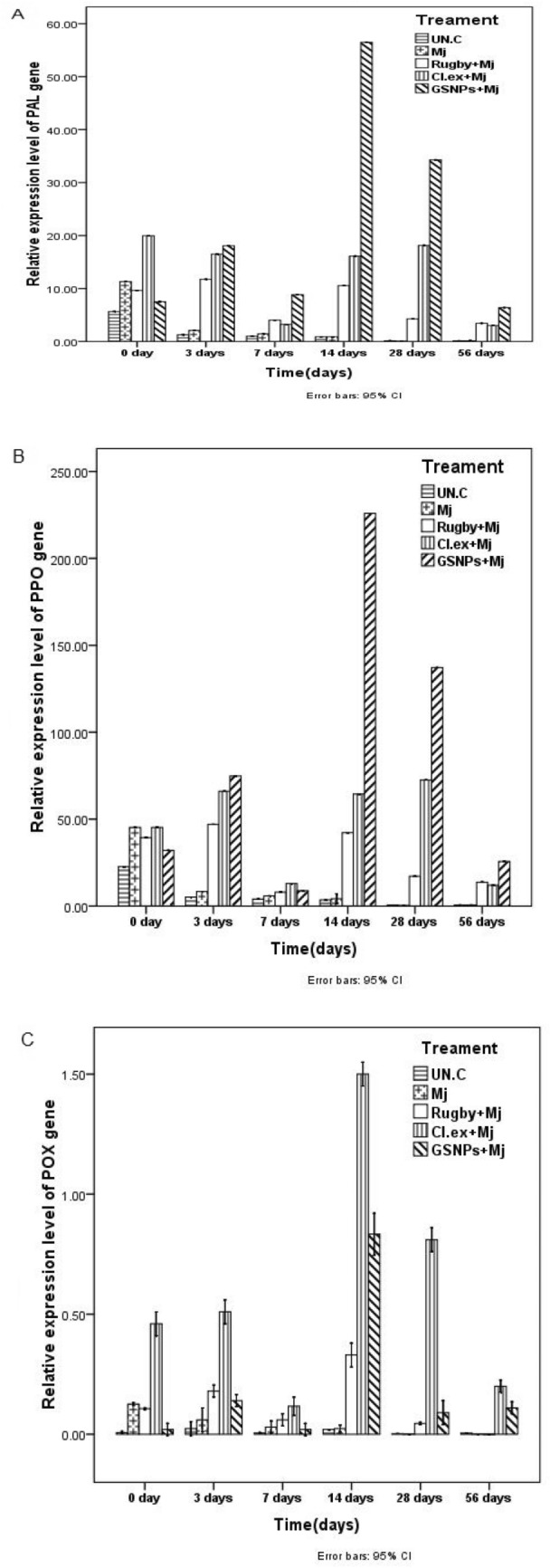
Quantitative Real-Time Polymerase Chain Reaction (qRT-PCR) validation of (**A**) *PAL*, (**B**) *PPO*, and (**C**) Pox relative genes expression in roots of tomato plants inoculated with *M. javanica* (Mj) and treated with *C. glomerata* extract, GSNPs with S/2 and the nematicide Rugby 60%. Untreated un-inoculated plants served as control (Un.C). The expression level of the target genes was normalized relative to the actin rRNA gene and relative expression of untreated control plants at each time. Each value represents mean ± S.E (n = 3).

The real-time PCR comparative technique was utilized in the quantification analysis, which mathematically transforms the Cycle Threshold (CT) into the relative expression level of genes. The relative expression level of the three defense genes, Phenylalanine Ammonia Lyase (*PAL*), Poly Phenol Oxidase (*PPO*), and Peroxidase (*POX*) were quantified by quantitative real-time PCR in the roots of tomato plants inoculated with *M. javanica* and treated macroalgal extract *C. glomerata*, GSNPs, Rugby nematicide, in addition to the untreated plant (Control). The expression profile of these genes was analyzed at 0, 3, 7, 14, 28, and 56 DANI (see Fig. 8). The results showed that the high expression level of the *PAL* gene was noticed in the infected plants treated by GSNPs and *C. glomerata* extract with 56.5 and 16-fold change, respectively, after 14 DANI when compared with the control (Mj alone) and Rugby 60% treatment (Fig. 7). These results clarified that overexpression of the *PAL* gene is directly dependent on pathogen stimulation at the first days post inoculation with a root-knot nematode (*M. javanica*). Current results indicate that a GSNPs with 75% concentration and *C. glomerata* extract can induce systemic acquired resistance. These observations confirm that JA plays an important role in *PAL* enzyme mediation which leads to the increase in the cell wall lignification as a physical barrier to phytopathogenic agents. However, the high expression level of the *PPO* gene was noticed after 14 and 28 DANI in plants treated with GSNPs followed by *Cladophora* extract at 28 and 14 DANI post inoculation, respectively, compared with control (Fig. 7A,B). On the other hand, the low expression level of the pox gene was recorded in tomato plants treated with all treatments (Fig. 7C). In our study, control plants (Untreated) showed no significant increase in *POX* activity whereas an increase of the expression level of *PAL* and *PPO* genes was found at different treatments during all times-points after inoculation (Fig. 7). These data suggest that fundamental role of GSNPs on activation, upregulation of defense-related genes, and induction of defense against the infection of root-knot nematodes (*M. javanica*). Ag-NPs were found to exhibit significant antimicrobial activity. The general notice is that the Green Synthesized Nanoparticles (GSNPs) induced the plant defense genes which rustled in high-rate production of the enzymes controlled by these genes. The high productions of these enzymes are considered as one of the acquired resistances for the plant against the plant pathogen.

## Discussion

This investigation aimed to investigate the efficacy of algal extract and their silver nanoparticles (GSNPs) as eco-nematicide against root-knot nematode (*Meloidogyne javanica*) infecting tomatoes. Moreover, the efficacy of algal extract and their GSNPs on expression-defending gene change of tomato was studied using different specific primers, i.e., *PAL*, *PPO*, and *POX*. The current results include the morphological characteristics of *C. glomerata* that clarified algal filament was a branched chlorophyte with large cylindrical cells forming long, regularly branched structures as reported before by^45^. The observed dark brown color indicated the formation of silver nanoparticles. Such a color transformation is mainly referring to changes in the oxidation state of metals. We observed that Ag^+^ was reduced to Ag by some biomolecules in algal extracts. Consequently, the reaction time increasing the reaction rate was gradually increased as shown by^46,47^ who reported that the appearance of dark brown color may be due to the excitation of Surface Plasmon Resonance (SPR) effect and reduction of AgNO_3_. Also, the formation of Ag-NPs was confirmed by visual assessment. This absorbance peak indicated a Surface Plasmon Resonance (SPR). Similarly^15^, it was demonstrated that the absorbance has occurred at 480 nm wavelength and has great slope specifies for the formation of nanoparticles. Various metals nanoparticles ranged from 2 to 100 nm in size, which has already been shown by^48^.

The FT-IR spectrum was used to characterize the possible biomolecules responsible for the reduction of the silver ions of the *C. glomerata* biosynthesized nanoparticles Ag-NPs (GSNPs). The results showed the presence of prominent and distinct peaks (3404, 2933, 2546, 2150, 1641, 1448, 1076, and 468) and these results were agreeing with^49,50^. Consequently, the FT-IR results imply that the Ag-NPs were successfully synthesized and capped with biocompounds present in the *C. glomerata* extract by using a green method^51^. Hence, the spectrum of *C. glomerata* aqueous extract was consistent with a sulfated polysaccharide, which is related to its antioxidant activity. This is like the results yielded by ^52,53^, who reported that the higher sulfate content of polysaccharides from marine algae exhibited stronger antioxidant activity. To study the morphologies of biosynthesized SNPs, the SEM analysis of colloidal was in spherical and hexagonal form Ag-NPs. The results were within 15–50 nm in sizes and some of them were in the form of agglomerates. GSNPs, likewise^31^, were maintained particle sizes between 10 and 50 nm in cubic and hexagonal shape synthesized by *Argemone mexicana* leaf extract. Similarly^54^, found that the particle size between 25–44 nm with cubical and hexagonal forms synthesized by freshwater green alga *Pithophora oedogonia*.

In our study, tomatoes infected with *M. javanica* showed significantly different physiological responses when treated with GSNPs (S, S/2) and *Cladophora* extract in a laboratory experiment (bioassay), where it yielded about 100% reduction in eggs hatching experiment at 48 h and also reached a rate of 100% after 24 h for mortality of J2S (see Tables 2 and 3), respectively. Thus, these results suggest that our GSNPs can be strongly nominated as eco-nematicide against root-knot nematode *M. javanica*. These results may be due to some secondary metabolite products such as the hormone components, indoles, cytokinins, gibberellins, brassinosteroids, and other compounds, which are considered as plant growth regulators including amino acids, peptides, and polyamines in amphora in higher concentrations than in spirulina. This conclusion is consistent with that of^55^, in addition to a higher amount of different antimicrobial and nematicidal functions such as being antioxidants, polyphenols, flavonoids, and some enzymes as recorded by^56^. Besides, it promotes plant growth and health by increasing shoot and root systems. The root is the primary gate through which nematode attacks and penetrates, causing infection and plant damage.

*PAL* is the main enzyme of phenylpropanoid metabolism and is closely linked to the production of phenolic, coumaric, and caffeic compounds such as toxic chemical substances during nematode infection^38,57–59^. Our study proposed that the induction of *PAL* and *PPO* activities was significantly higher in infected tomato plants after treated with algal extract and GSNPs, respectively, than in the non-inoculated and untreated ones (see Fig. 8). This was consistent with the observation regarding the interrelation between defense systems in tomato plants and *M. javanica*. Current results clarified that overexpression of the *PAL* gene is directly dependent on pathogen stimulation at the first days post inoculation with a root-knot nematode (*M. javanica*). These results indicate that a GSNPs with 75% concentration and *C. glomerata* extract can induce systemic acquired resistance. These observations confirm that JA plays an important role in *PAL* enzyme mediation which leads to increase cell wall lignification as a physical barrier to phytopathogenic agents^12,60^. Besides, phenolic compounds are catalyzed by *PPO* into anthraquinones or terpenoids, which can inhibit the invasion of pests and diseases. *POD* is responsible for multiple crosslinking of cell wall lignins, and lignins act as potential physical barriers against nematodes.

In our study, control plants showed no significant increase in *POX* activity whereas an increase of the expression levels of *PAL* and *PPO* genes was found at different treatments during all times-points after inoculation. These results agreed with^26,61^; the relation between *PPO* enzymes to wounding and enzymatic browning had been studied in several plants. And this enzyme plays an important role in the biosynthesis of alkaloids under biotic and abiotic stress^62^. Lignification confers mechanical strength to plant cell walls to enhance host defense against pathogenic invasion^63–65^. These data suggest that the fundamental role of GSNPs on activation is the upregulation of defense-related genes and induction of defense against the infection of root-knot nematodes (*M. javanica*). Ag-NPs were found to exhibit significant antimicrobial activity. Ag-NPs have great potential to act as antimicrobial agents^66^. The general notice is that the Green Synthesized Nanoparticles (GSNPs) incited the plant defense genes which resulted in high-rate production of the enzymes controlled by these genes^67^. The high productions of these enzymes are deemed as one of the acquired resistances for the plant against the plant pathogen and these results agree with that of^68^. On the other hand, it is well known that polyphenols are produced in high amounts in plants infected with nematode due to the high expression of their genes^69^.

## Conclusions

It can be concluded that tomato plants infected with root-knot nematodes (*M. javanica*) showed significant physiological responses and promoted plant growth and health by increasing shoot and root systems while treated with our GSNPs. Also, it induced the excitation of the immune responses of plants against infection with the nematode. We suggest that GSNPs can be strongly nominated as an eco-nematicide against root-knot nematode *M. javanica*. It is also known that the root is the primary gate through which nematode attacks and penetrates, causing infection and plant damage.
